# Diverse roles of connexins in the neuro-vasculature in homeostasis and disease

**DOI:** 10.1515/tnsci-2025-0400

**Published:** 2026-07-09

**Authors:** Valentin Delobel, Nafiisha Genet, Karen K. Hirschi

**Affiliations:** Department of Cell Biology and Developmental Genomics Center, University of Virginia School of Medicine, Charlottesville, VA, USA; Robert M. Berne Cardiovascular Research Center, University of Virginia School of Medicine, Charlottesville, VA, USA

**Keywords:** gap junctions, connexins, neurons, astrocytes, blood-brain barrier

## Abstract

Significant progress has advanced our understanding of brain physiology. The brain is now recognized as an integrated network rather than a compartmentalized organ, composed of diverse cell types including neurons, glial cells, and vascular components. Communication within this neurovascular unit relies on multiple receptors, transporters, and channels. Among these, gap junction channels formed by connexin proteins play a central role by enabling direct intercellular exchange of ions, metabolites, and signaling molecules. In the healthy brain, connexins contribute to homeostatic balance and neuronal activity, whereas their dysregulation has been implicated in the pathogenesis of multiple neurological disorders. In this review, we summarize current knowledge on the physiological roles of connexins in the brain, analyze evidence from preclinical and clinical studies linking connexin dysfunction to disease, and discuss emerging therapeutic strategies targeting connexin-mediated signaling for the treatment of brain disorders.

## Introduction

Organs are composed of diverse cell types arranged in a highly organized manner that ensures proper function and maintenance of tissue homeostasis. Effective cell–cell communication is essential to coordinate activities across the different layers of this complex hierarchy. In the brain, neuronal activity is supported and protected by glial cells including astrocytes, oligodendrocytes and microglia. To sustain this highly metabolically active organ, reciprocal interactions between glial and vascular cells are essential. The so-called neurovascular unit plays critical roles in the brain by delivering nutrients and oxygen and removing waste products, while providing a barrier that protects the brain parenchyma from neurotoxic and pathogenic components that could compromise neuronal activity.

Gap junctions (GJ) directly connect the cytoplasm of two adjacent cells, forming intercellular channels that allow the passage of small molecules and ions of approximately 1 kDa or less. These channels are composed of proteins called connexins which are ubiquitously expressed by cell types in the brain. Under physiological conditions, the regulated function of GJ channels sustains neuronal activity and brain homeostasis. However, under pathological conditions, abnormal GJ activity can impair vascular barrier function and promote the release of neurotoxic molecules or pro-inflammatory factors, thereby contributing to the progression of prevalent neurodegenerative diseases, such as Alzheimer’s disease (AD), Parkinson’s disease (PD) or Multiple Sclerosis (MS).

In this review, we will first summarize the expression pattern of connexin proteins in the brain and their roles in maintaining homeostasis. We will then examine how connexin dysregulation contributes to central nervous system (CNS) pathophysiology and highlight current therapeutic strategies targeting connexins in neurological disorders.

## Gap junctions: structure, assembly and regulation

### Connexin structure and gene family

**Figure 1:**
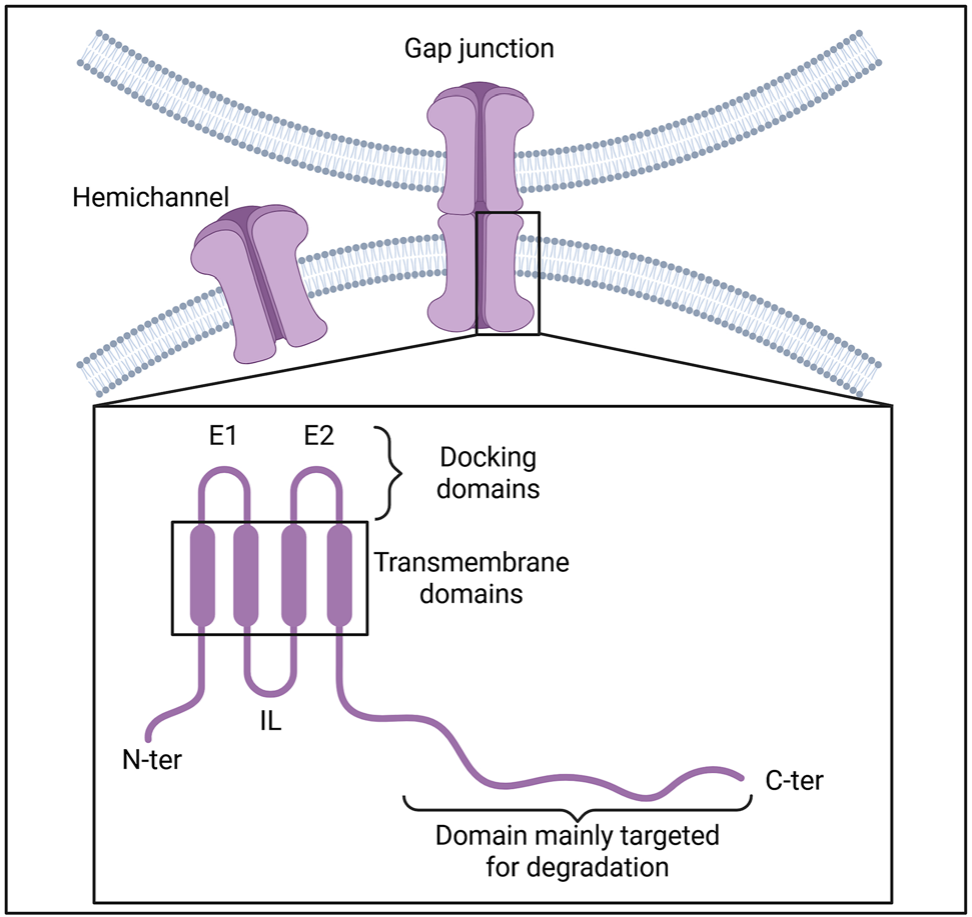
Structure of connexin proteins, hemichannels and gap junctions.

Six connexins assemble into a hexameric unit known as a connexon, or hemichannel, which anchors within the plasma membrane. Two hemichannels, one from each adjacent cell membrane, form GJ channels [1], 2]. To date, 21 connexin genes have been identified in humans and 20 in mice [3], 4]. Despite this diversity, all connexins share a highly conserved structure consisting of four transmembrane (TM) domains (TM1–TM4), two extracellular loops (E1 and E2), and three intracellular regions: an intracellular loop (IL), and the N- and C-terminal domains [1] (Figure 1).

### Gap junction assembly and docking

Connexin proteins are synthesized in the endoplasmic reticulum (ER), where they begin oligomerization into hemichannels. These are subsequently transported in trafficking vesicles along the microtubule network to the plasma membrane [5]. Hemichannels may be homomeric (composed of identical connexins) or heteromeric (composed of different connexins). At the plasma membrane, hemichannels from adjacent cells can dock together to form a transmembrane pore, resulting in a functional GJ channel [6], 7]. Docking specificity is regulated by the extracellular loops E1 and E2, which determine compatibility between connexins [8]. For example, connexin 46 (Cx46) can form heterotypic GJs with Cx43 and Cx50, whereas Cx50 and Cx43 cannot. Chimeric studies replacing the E2 domain of Cx46 with that of Cx50 abolished its ability to pair with Cx43, confirming the critical role of E2 in docking compatibility [7]. Structural insights from crystallographic studies of Cx26, one of the most amenable connexins to crystallographic analysis, further revealed conserved motifs within E1 and E2 involved in hydrogen and disulfide bond formation [6], [9], [10], [11]. Mutations within these residues disrupt docking and prevent channel formation. Finally, it is possible that a hemichannel does not dock to another one on an adjacent cell. However, it can still function in an open state (non-junctional configuration), notably for the release of cytokines [12], and gliotransmitters, such as ATP or glutamate [13], 14].

#### Hemichannels

Connexins can function either as intercellular GJ channels or hemichannels. GJ channels support direct cell–cell communication required for coordinated ionic and metabolic homeostasis, whereas hemichannel opening can mediate the release of ATP and other extracellular signaling molecules [15], [16], [17], [18]. It is important to distinguish these two configurations when considering the roles of connexin proteins in CNS physiology and disease.

### Connexin turnover and degradation

GJs are highly dynamic structures with relatively short half-lives ranging from 1 to 10 h, depending on connexin and tissue type [19]. This rapid turnover contrasts with other channel-forming proteins that often persist for days. Connexins targeted for degradation are internalized into double-membrane vesicles, termed connexosomes, which can be visualized using cysteine-tagged recombinant proteins and electron microscopy [20]. Connexosomes are trafficked to endolysosomal and phagolysosomal pathways for degradation [2], [21], [22], [23]. Post-translational modifications tightly regulate connexin degradation. Phosphorylation of residues, particularly within the C-terminal domain, promotes internalization and degradation. Kinases, such as mitogen-activated protein kinase (MAPK), protein kinase C (PKC), p34, and Src have been implicated in this process [18], [22], [23], [24]. Alternatively, connexins can undergo ubiquitination, targeting them for proteasomal degradation [18], 25].

### Cellular functions of connexins and emerging therapeutic strategies

#### Channel function

The channel function of connexin proteins regulates multiple cellular functions, such as calcium influx [26], 27], metabolism [28], proliferation and migration [29], permeability [30], and neuronal activity [31], [32], [33].

GJs and hemichannels permit the diffusion of small molecules of less than ∼1 kDa, including ions, ATP, small peptides, nutrients and nucleic acids [1]. Their permeability depends on the dynamic opening and closing of the hemichannels. Each hemichannel can close through the assembly of its N-terminal domains, which form a plug that obstructs the channel pore [34]. For a channel to open, both hemichannel plugs must retract to allow passage of ions and signaling molecules; however, closure of a single hemichannel is sufficient to block the intercellular junction. Cryo-electron microscopy studies have identified an intermediate “semi-closed” or semi-permeable state, which likely contributes to channel selectivity [35]. In the closed conformation, the plug is stabilized by a network of hydrogen bonds and hydrophobic interactions. GJ gating can be triggered by diverse stimuli, including changes in voltage, Ca^2+^ fluxes, or membrane polarization [36], [37], [38], [39]. Computational models have demonstrated how connexin gating contributes to cardiac excitability and reduces the risk of fibrillation [40]. Additional modulators include pH changes, environmental stresses, and post-translational modifications, such as connexin protein phosphorylation [41], [42], [43], [44], [45].

Traditionally, GJs were considered permeable only to molecules ≤1 kDa. However, this paradigm has been challenged by recent findings showing that microRNAs (miRNAs) can diffuse through GJs [46]. This discovery has opened new avenues for therapeutic strategies that harness connexins as conduits for nucleic acid-based delivery between cells. For instance, connexosome-like vesicles have been engineered to deliver dextran molecules of up to 10 kDa into the cytosol, demonstrating the potential of connexins as carriers for larger therapeutic cargo [47].

Importantly, permeability is connexin protein dependent. Cx43-based channels in particular show greater capacity to mediate transfer of molecules >1 kDa and display enhanced permeability to small interfering RNAs (siRNAs) compared with Cx26 or Cx32 channels [46], [47], [48]. These observations highlight the possibility of selectively targeting specific connexins to optimize intercellular drug delivery or gene therapy approaches.

#### Non-channel function

Connexins are also involved in intracellular molecular cascades to regulate various cellular functions independently of their channel function. Studies demonstrated that the C-terminal tail of connexins is involved in multiple signaling pathways regulating cell proliferation, migration or differentiation. For example, Cx37 has been shown to promote growth arrest of endothelial cells in G1 phase of cell cycle by sequestrating Extracellular signal-Regulated Kinase (ERK) in the cytoplasm in order to prevent Forkhead box O3a (Foxo3A) degradation, a transcription factor that induces expression of Cyclin-Dependent Kinase Inhibitor 1B (*CDKN1B*), which encodes cell cycle inhibitor, p27 [49]. Cx43 also plays a role in neuronal development in a channel-independent manner, involving both ERK and p27. It was shown that Cx43 regulates p27 expression to control neuronal multipolar phases during their migration from the ventricular zone towards the cortex during brain development [50]. Our group demonstrated that, in the subventricular zone, one of the largest neural stem cell (NSC) niches in the adult brain, Cx43-mediated interactions between NSC and endothelial cells maintain NSC quiescence and survival, in a channel-independent manner, involving activation of ERK signaling through the Cx43 cytoplasmic tail [51].

## Gap junctions in brain homeostasis

### Expression of connexins in the central nervous system

**Figure 2:**
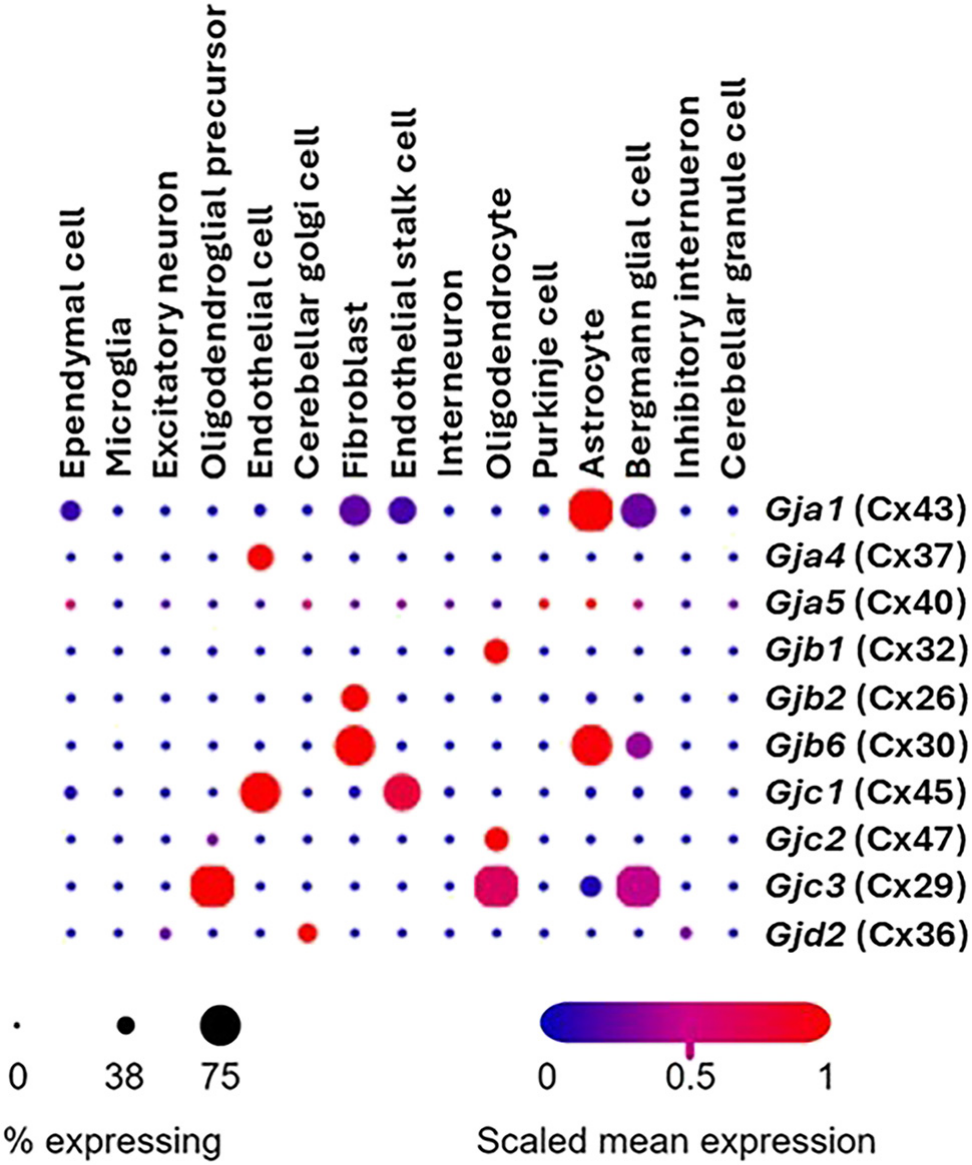
Relative connexin transcript expression in cerebellar cell types.

**Figure 3:**
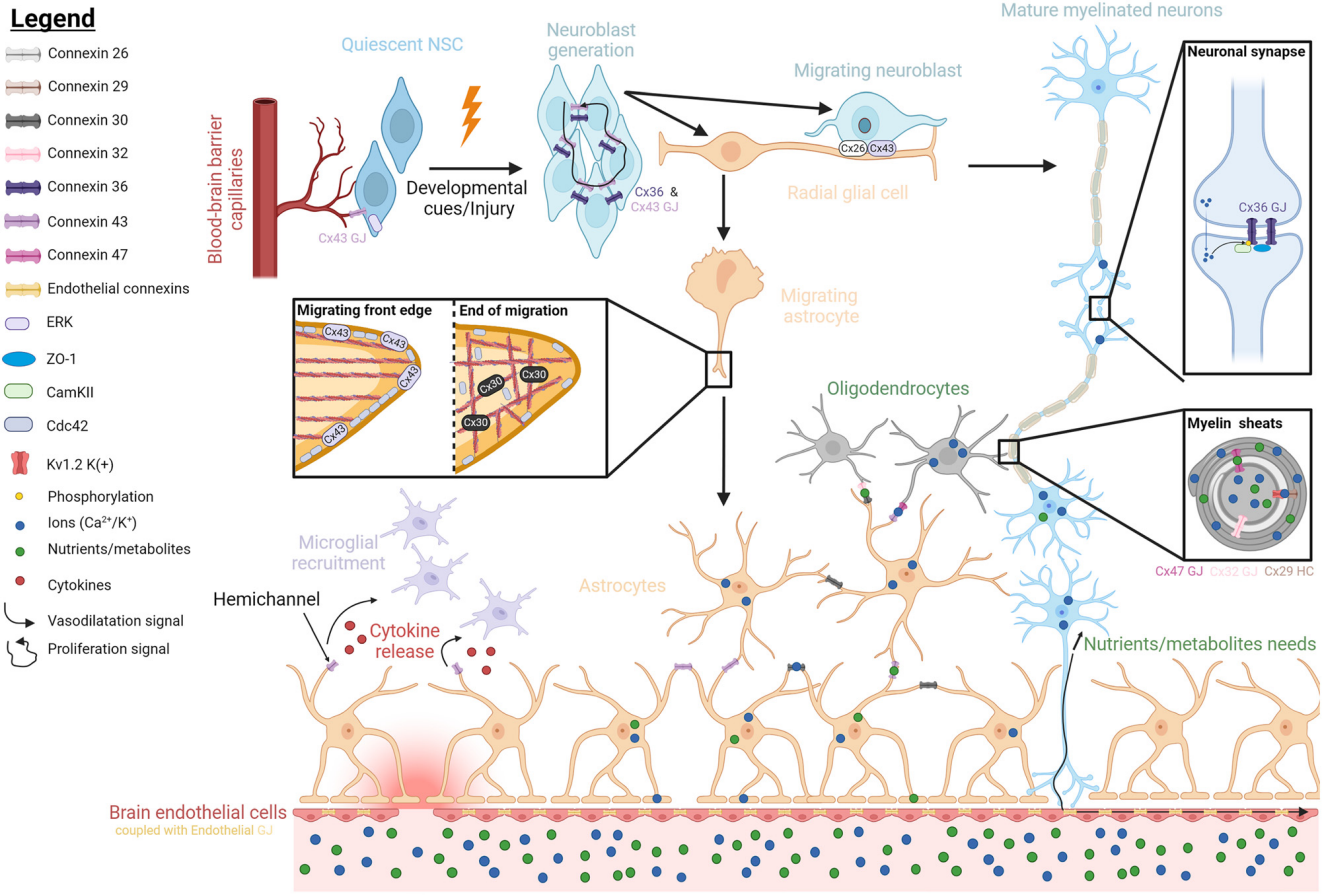
Physiological roles of connexins in the brain. Interactions NSCs and ECs through Cx43-mediated GJs regulate neuroblast generation. During neuroblast migration, Cx36-based GJs facilitate communication along radial glial cells, while astrocytic migration is enabled by Cx43, and migration arrest involves Cx30, which contributes to cytoskeletal disassembly. In mature neurons, Cx36-GJs predominate at synapses, supporting electrical and metabolic coupling. Oligodendrocyte-axon interactions are maintained through Cx47-GJs, Cx32-GJs, and Cx29-hemichannels (HCs), allowing the exchange of ions and metabolites essential for neuronal homeostasis. Within the cerebral vasculature, ECs are interconnected via GJs that enable the propagation of vasodilatory signals, thereby coordinating neurovascular coupling and blood flow regulation.

Using a published single-cell RNA sequencing dataset of gene expression in wild-type adult mouse cerebellum, combined with the Single Cell Portal, we generated a dot plot highlighting the distribution of connexin transcripts across CNS cell populations (Figure 2) [52]. We show that distinct connexins are enriched in specific cell types, with *Gja4* (Cx37) and *Gjc1* (Cx45) predominating in endothelial cells, *Gjb1 (Cx32)* and *Gjc2 (Cx47)* enriched in oligodendrocytes [53], [54], [55], and *Gja1* (Cx43) and *Gjb6* (Cx30) predominantly expressed in astrocytes [56], 57]. These expression profiles are consistent with the specialized functions of connexins across the major brain cell types, including neurons, astrocytes, oligodendrocytes, neural stem cells, and vascular cells. The known physiological roles of connexins in brain homeostasis are summarized in Figure 3, while Table 1 provides the corresponding references for these cell type-specific functions and their disease associations.

**Table 1:** Connexin genes, expression, and disease associations.

Connexin, gene	Main expression sites	Role(s) in homeostasis conditions	Dysregulation/disease association	References
Cx26, GJB2	Neurons, astrocytes	Adhesion of neurons to radial glial cell for neuronal migration in neocortex; astrocyte differentiation	X	[58], [59], [60], [61]
Cx29, *GJC3*	Oligodendrocytes	Supplying myelin sheath with ions	X	[62], 63]
Cx30, *GJB6*	Astrocytes	Astrocyte differentiation; Astroglial synapse invasion; Propagating ions and metabolites for sustaining neuronal activity; BBB integrity via astrocyte endfoot organization; pericyte marker	Modulated in Parkinson’s disease	[56], 59], [64], [65], [66], [67], [68], [69], [70], [71], [72], [73], [74]
Cx32, *GJB1*	Oligodendrocytes	Myelin support; oligodendrocyte–axon communication	Multiple sclerosis; demyelination	[75], [76], [77], [78], [79], [80]
Cx36, *GJD2*	Neurons (interneurons, excitatory neurons)	Synchronization of neuronal firing; synaptic plasticity	Epilepsy	[81], [82], [83], [84], [85], [86], [87], [88]
Cx37, *GJA4*	Endothelial cells; smooth muscle cells	Arterial identity; vascular tone	X	[89], 90]
Cx40, *GJA5*	Endothelial cells	Endothelial conduction; vasomotor responses	X	[89], 91]
Cx43, *GJA1*	Astrocytes, endothelial cells, NSCs, meninges	Neurogenesis; migration on radial glial cell; maintenance of neural stem cell pool; post-injury neurogenesis; Astrocyte differentiation; propagating ions and metabolites for sustaining neuronal activity; brain inflammation and repair; oligodendrocyte-astrocyte communication; astrocyte–astrocyte and astrocyte–endothelial coupling; BBB stability;	Neuroinflammation (AD, MS, PD…); glioblastoma progression; cerebral hypertension, epilepsy; loss of BBB integrity during aging	[12], 28], 50], 51], 61], 64], 65], [72], [73], [74], [75], [89], [91], [92], [93], [94], [95], [96], [97], [98], [99], [100], [101], [102], [103], [104], [105], [106], [107], [108], [109], [110], [111], [112], [113], [114], [115], [116]
Cx47, *GJC2*	Oligodendrocytes	Myelin production; sustaining neuronal activity with nutrients; Oligodendrocyte–astrocyte coupling;	Pelizaeus–Merzbacher–like disease; demyelination	[76], [77], [78], [80], [116], [117], [118]
Cx45, *GJC1*	Endothelial cells	Vascular identity; regulation of electrical coupling	X	[89]

Although connexins participate broadly in multiple aspects of CNS biology, this mini-review focuses specifically on their roles in the neurovascular unit, with particular emphasis on blood-brain barrier function and neurodegenerative diseases.

### Neurovascular unit and blood-brain barrier

The blood-brain barrier is a highly selective interface that separates blood circulation from the brain parenchyma, protecting neural homeostasis from neurotoxic substances [119]. Structurally, it consists of a specialized endothelium characterized by high expression of tight junction proteins, selective transporters, lack of fenestrations and low rates of transcytosis [120], [121], [122], [123]. Mural cells form the supporting vessel wall and include smooth muscle cells that surround large arteries and veins, and pericytes that cover capillaries. In the blood-brain barrier, the final component of this complex structure is astrocytic end feet, also called glia limitans, which contact the vasculature [124], 125]. A perivascular space between astrocytes and mural cells permits the flow of cerebrospinal fluid and provides a niche for other cell populations, including macrophages and perivascular fibroblasts [126], 127].

Connexins play an important role in the blood-brain barrier by regulating the delivery of nutrients and metabolites, notably glucose and pyruvate, to neurons. Substrates entering from the bloodstream are exchanged via selective transporters to glial cells, which then relay them to neurons, largely through GJ-coupling, as discussed above.

The neurovascular unit, which integrates interactions between neurons, glia and the vasculature, tightly regulates nutrient supply. In response to increased neuronal activity, relaxation of mural cells induces vasodilation and increased cerebral blood flow [128], 129]. The vasodilation signal propagates through GJs distributed throughout the brain vasculature. One study mapped a gradient of connexin protein expression, along the vascular continuum: Cx40 is exclusively expressed in arteries while Cx37 is found in arteries and arterioles. Cx43 is expressed at arteriole-capillary and capillary-venule transitions, and Cx45 is present in capillaries, venules, and veins. The arterial connexins are thought to initiate and coordinate vasodilation, transmitting hyperpolarizing signals through the endothelium to match capillary blood flow with neuronal energy demand. Indeed, genetic deletion of both Cx37 and Cx40 slows and attenuates this signaling, resulting in reduced vasodilation [89].

Moreover, connexins protect the brain parenchyma and maintain neuronal activity by supporting the structural integrity of the blood-brain barrier. In the brain, endothelial connexins, including Cx40 and Cx43, participate in junctional complexes together with tight-junction proteins, such as ZO-1, Occludin, and Claudin-5. Through their C-terminal PDZ-binding motifs, connexins interact with the scaffolding protein ZO-1, which in turn anchors tight-junction components. Claudin-5 serves as the primary transmembrane protein responsible for paracellular sealing at the blood-brain barrier, whereas Occludin plays a central role in stabilizing barrier architecture [91], 92].

At glial limitans, the Cx43 C-terminal stabilizes astrocytes. In mouse, global deletion of Cx30 and Cx43 or truncation of the Cx43 carboxyl tail leads to increased mobility of GJ plaques, loss of Aquaporine-4 (AQP4), and microhemorrhages [64], 65]. Aging is likewise associated with decreased blood-brain barrier function. In 19 month-old mouse brains, gene expression for barrier components including *Cldn5*, *Mfsd2a*, *Slc2a1*, and *Tfrc* are downregulated, whereas genes linked to non-specific vesicular transport (*Cav1*, *Cav2*) are upregulated [130]. Recent work further shows that *Gja1* (encoding Cx43) expression is reduced in the cortex of mice aged between 18 and 22 months-old, associated with blood-brain barrier leakage and cognitive decline. Mechanistically, loss of Cx43 triggers mitophagy and endothelial senescence, resulting in barrier disruption. Notably, channel-independent functions of Cx43 preserve NAD^+^ levels in blood-brain barrier endothelial cells, sustain mitochondrial activity and maintain barrier integrity [93]. Since connexin expression is downregulated with aging, we can hypothesize that neurovascular coupling, and thus activity-dependent glucose delivery, is impaired, contributing to age-related cognitive decline.

Inflammation is another factor that compromises the integrity of the blood-brain barrier by promoting the opening of hemichannels and GJs. During neuroinflammatory responses, pro-inflammatory cytokines, such as IL-1β and TNF-α, secreted by activated microglia triggers calcium waves, that, in turn, open Cx43 hemichannels and GJs [94]. *In vitro* studies have shown that Gap27, an inhibitory peptide targeting Cx43, attenuates calcium-wave-dependent opening of these channels. Similarly, blockade of Calcium/Calmodulin-Dependent Protein Kinase II (CamKII) reduces the blood-brain barrier permeability [131], 132]. In mouse, lipopolysaccharide (LPS)-induced inflammation or intracerebral hemorrhage injury enhances astroglial and endothelial Cx43 expression and increases hemichannel opening, facilitating the release of pro-inflammatory cytokines, such as IL-1β, IL-6, TNF-α or IFN-γ [12], 95]. Based on its important role in blood-brain barrier dysfunction, Cx43 is considered a promising therapeutic target for neuro-inflammatory and systemic inflammatory disorders via the use Cx43-specific peptides like Gap19 or Gap26/27. This topic will be discussed in Section 4.

Although the roles of connexins in vascular beds have been extensively investigated, their contribution to the development and function of the blood-brain barrier remains understudied [3]. For example, our group has demonstrated that in postnatal mice, Cx37 is a key regulator of arteriovenous differentiation, via a cell cycle control mechanism. Specifically, arterial shear stress activates NOTCH signaling which, in turn, upregulates Cx37. Mechanistically, Cx37 sequesters ERK in the cytoplasm, preventing the silencing of *CDKN1B*, which encodes p27, a cyclin-dependent kinase inhibitor that promotes endothelial cell cycle arrest, which enables arterial-venous gene expression [49], [133], [134], [135]. Single cell RNA sequencing analyses of the adult mouse brain indicate that Cx37 expression is enriched in arterial endothelial cells within the blood-brain barrier [90]. Moreover, loss of canonical NOTCH signaling in endothelial cells results in cerebral arteriovenous malformations, which is associated with downregulation of arterial markers and increased expression of venous markers [136]. These findings suggest that Cx37 may participate in blood-brain barrier arteriovenous specification, and its disruption could potentially contribute to the formation of brain arteriovenous malformations, which requires further investigation.

Interactions between mural cells and other cellular components of the blood-brain barrier via connexins is poorly studied. Smooth muscle cells regulate cerebral blood flow through their contractile activity thereby contributing to blood-brain barrier integrity. Chronic systemic hypertension, a recognized risk factor for neurodegenerative disorders, impairs barrier function. Notably, one study demonstrated that Cx43 is upregulated in smooth muscle cells during Angiotensin-II-induced hypertension. Under these conditions, Cx43 promotes smooth muscle cell proliferation through upregulation of the MAPK pathway, leading to enhanced contractility of brain arterioles [96]. These observations suggest that Cx43-targeting peptides may have therapeutic potential for limiting blood-brain barrier dysfunction and associated CNS pathology, although this possibility remains to be tested more rigorously.

Despite these insights, studies examining the roles of connexins and GJs in brain mural cells are lacking. As mentioned earlier, Cx37 is expressed not only in arterial endothelial cells but also in smooth muscle cells within the blood-brain barrier [90], raising the possibility of heteroceullular Cx37 GJs mediating communication between smooth muscle and endothelial cells. Furthermore, Cx30 has been identified as a marker of a subset of pericytes [66], although its precise function therein is still unknown. Additional research is therefore needed to elucidate the contribution of connexins, namely Cx37 and Cx30, in mural-endothelial cell–cell interactions in blood-brain barrier development, maintenance, and pathology.

## Gap junctions and neurovascular anomalies

### Gap junction dysfunction in neurological disorders

Although connexin-targeted interventions have attracted growing interest in CNS disease, their translational development remains at an early stage. At present, most proposed strategies are supported by preclinical evidence, whereas clinical data remain limited. Accordingly, we discuss the examples below as emerging therapeutic avenues rather than clinically established approaches.

#### Neuroinflammation

Neuroinflammation is increasingly recognized as a key risk factor for cognitive decline and dementia. Recent studies have highlighted its association with blood-brain barrier dysfunction, disruption of cerebral network integrity, and cognitive decline [137], [138], [139]. Consequently, numerous therapeutic strategies have been proposed to mitigate neuroinflammation and preserve blood-brain barrier integrity [140], [141], [142]. Connexins, expressed in both endothelial cells and astrocytes, are central to blood-brain barrier maintenance, and their dysregulation exacerbates neuroinflammatory processes. In neuroinflammation and neurodegeneration, it is important to distinguish pathological hemichannel opening from GJ channel coupling, as these two connexin configurations have different effects on inflammation and blood-brain barrier integrity.

Inflammatory stimuli disrupt GJ plaques and promote hemichannel opening, leading to the release of pro-inflammatory cytokines, loss of barrier function, and astrogliosis [12], 64]. Genetic studies in mice, using *Gja1* (encoding Cx43) conditional knockouts in murine astrocytes or endothelial cells demonstrate that Cx43 deletion in either cell type restores blood-brain barrier integrity and reduces cytokine release in lipopolysaccharide (LPS)-induced inflammation [12]. Pharmacological approaches targeting disruption of Cx43 function further support its potential as a therapeutic target to prevent neuroinflammation. For example, under inflammatory conditions, intravenous administration of Cx43-blocking peptides, such as Gap27 or Gap19, reduces inflammatory responses and limits blood-brain barrier leakage. Additionally, the use of Ca^2+^ chelators, such as 1,2-bis(o-aminophenoxy)ethane-N,N,N’,N’-tetraacetic acid acetoxymethyl ester (BAPTA-AM), has also been proposed to inhibit Cx43 channel and hemichannel activity, thereby reducing neuroinflammatory signaling [12]. The use of Gap19 in neuroinflammation is promising as it’s modified form, TAT-Gap19, crosses the blood-brain barrier and selectively inhibits Cx43 hemichannels without impairing astrocytic GJ coupling, preserving glial support for neuronal ionic and metabolic homeostasis [143]. In a model of intracerebral hemorrhage, TAT-Gap19 reduced hemichannel opening and inflammation, partly via activation of the Yes-associated protein (YAP) signaling pathway, which suppressed TLR4–NFκB and JAK2–STAT3 cascades; these effects were reversed by a YAP inhibitor [95]. Similarly, small molecules, such as D4, a selective hemichanggel blocker, have shown efficacy in attenuating neuroinflammation in temporal lobe epilepsy [97].

While most therapeutic approaches focus on blocking Cx43-composed GJs or hemichannels to limit inflammation and barrier leakage, several studies highlight the potential benefits of restoring or enhancing Cx43 function during post-ischemic repair. Cx43 has also been identified as a mediator of exosome communication with target cells through protein kinase A (PKA)-dependent phosphorylation [144], 145]. In an ischemia-reperfusion rat model, Chen and colleagues showed that activating PKA with 8-bromo-cAMP increased the release of Cx43^+^ exosomes into the cerebrospinal fluid; their uptake by astrocytes promoted cognitive recovery [98].

Together, these findings suggest that, in addition to inhibiting pathological channel activity, PKA-mediated phosphorylation of Cx43 may facilitate reparative exosome signaling and support post-ischemic brain function.

#### Dementia

Alzheimer’s disease (AD), a leading cause of dementia, is associated with increased blood-brain barrier permeability and the accumulation of amyloid-β (Aβ) plaques [146], [147], [148]. Post-mortem analyses of human brains have shown that 80 % of Aβ plaques have increased expression of Cx43 in astrocytes, suggesting a key role for Cx43 in AD pathology. Aβ deposits enhance astrocytic calcium waves, promoting hemichannel opening and further compromising the blood-brain barrier integrity. In addition, Aβ activates glial cells, stimulating ATP and neurotoxic glutamate release, which opens neuronal hemichannels and contributes to cell death. Genetic deletion of Cx43 in AD mouse models reduces neuronal loss, oxidative stress, astrocyte activation, and blood-brain barrier permeability [99], [149], [150], [151]. These findings highlight Cx43 as therapeutic targets for AD. Pharmacological blockade of Cx43-composed GJs with Gap26 or Gap27 improved learning and spatial memory in a rat model of ischemia, where Cx43 is upregulated at lesion sites [100], suggesting that similar approaches may benefit AD. Likewise, INI-0602, a hemichannel inhibitor that limits Ca^2+^ propagation, enhanced cognitive performance in APP/PS1 mice. INI-0602-treated animals showed better exploratory behavior in novel-object recognition, as well as improved learning in fear-conditioning and water maze tests. Notably, INI-0602 did not reduce Aβ plaques, aligning with evidence that cognitive improvement can occur independently of plaque clearance [152].

#### Parkinson’s disease

Parkinson’s disease (PD) is a progressive neurodegenerative disorder in which the hereditary forms (dominant or recessive) account for approximately 15 % of cases, whereas the majority are idiopathic, occurring from complex interactions between environmental and genomic factors [153]. Among environmental factors, neuroinflammation has been proposed as a key driver of idiopathic PD [154]. LPS-induced inflammation reproduces dopaminergic neuron degeneration in experimental models, mimicking PD pathology [155]. In such models, treatment with the Cx43 inhibitory peptide Gap27 rescued the LPS-induced drop in dopamine levels and improved damage to the bilateral dopaminergic pathway and attenuated microglia and astrocytes pro-inflammatory responses, thereby reducing cytokine release [101]. Evidence from post-mortem PD brains indicate that uncoupling of astrocytic Cx43 GJs correlates with areas of neuronal loss, highlighting the importance of distinguishing connexin hemichannel activity from GJ coupling. Mechanistically, Gap26 and Gap27 preferentially inhibit Cx43 hemichannels, with only prolonged exposure inhibiting GJs coupling. In contrast, TAT-Gap19 selectively blocks Cx43 hemichannels without altering GJ coupling and, importantly, crosses the blood-brain barrier, supporting its potential for future translational development [12], 143], 156]. Targeting hemichannel function while preserving GJ channel-mediated astrocytic coupling represents a promising therapeutic concept for PD; however, supporting evidence remains limited to preclinical studies.

In addition to Cx43, Cx30 has been described to play a protective role in the context of PD. In an inducible mouse model of PD, loss of Cx30 accelerated dopaminergic neuron degeneration by impairing anti-inflammatory A2 astrocytes, which are critical in dopaminergic neuron survival. A2 astrocytes express neuroprotective genes, such as *S100a10* and *Gdnf*, that are absent in Cx30-deficient PD mice. The authors also showed that deletion of Cx30 inhibits A2 astrocyte activation, assessed by lack of *Gfap* gene expression upregulation. With transcriptomic analyses, among pathways downregulated in Cx30-deficient PD mice, the authors observed dysfunctions in the axon-guidance signaling pathways, supported by a downregulation of axon-guidance ligands, namely ephrins, netrins and semaphorins [56]. Consequently, targeting astrocytic connexins may represent a relevant strategy for dopaminergic neuron protection, although this possibility requires further validation. Modafinil, a wake-promoting compound used to alleviate excessive daytime sleepiness in PD patients [157] has been shown to enhance astroglial GJ communication, notably by increasing Cx30 expression [67]. Two recent clinical trials (NCT03083132 and NCT02857244) evaluated Modafinil in PD and reported modest patient benefit; however, the findings remain limited, and the specific contribution of connexin modulation to these effects is still unclear [158].

#### Glial-related diseases

Multiple Sclerosis (MS), an autoimmune demyelinating disease in the CNS is among the most studied glial cell-related disorders. Altered expression of astroglial connexins has been reported in MS pathology. Clinical analyses of demyelinating lesions of post-mortem Japanese MS patients revealed the loss of heterotypic Cx43/Cx47 GJs between astrocytes and oligodendrocytes. Notably, the loss of Cx43 correlated with more severe disease progression, as 66.7 % of patients lacking Cx43 expression died within 2 years, whereas no death occurred in patients who retained Cx43 expression. In addition, patients with chronic MS lesions exhibited the loss of Cx32 expression, further implicating connexin dysfunction in disease progression [75]. Pre-clinical studies support these observations. In experimental autoimmune encephalomyelitis (MS mouse model), loss of Cx47 in oligodendrocytes was associated with severe neuroinflammation. Conversely, transplantation of mouse or human induced-pluripotent stem cells into a lysolecithin-induced demyelination model resulted in differentiation into Cx47^+^ oligodendrocytes capable of coupling with astrocytic Cx43, demonstrating their ability to integrate and restore intercellular communication within the brain parenchyma [159]. In the experimental autoimmune encephalomyelitis model, Cx43 expression is upregulated as a consequence of astrogliosis and is exacerbated by the loss of oligodendroglial Cx47 [55], 75], 102]. Specific deletion of *Gja1* (encodes Cx43) in murine astrocytes alleviated neuroinflammation by reducing immune cell infiltration and promoting neuroprotective A2-astrocytes [160]. Together, these findings identify Cx43 as a key regulator of neuroinflammation in MS, and pharmacological targeting of Cx43 is emerging as a therapeutic strategy. For example, Takase and colleagues demonstrated that treatment with the hemichannel inhibitor INI-0602 reduced neuroinflammation and demyelination in experimental autoimmune encephalomyelitis mouse model [161].

Connexins dysregulation is also observed in glioblastoma, a highly aggressive glial cell-related tumor that accounts for ∼50 % of malignant brain tumors. Despite treatment, 90 % of patients experience tumor recurrence within 5–7 months, resulting in poor overall survival [162]. Temozolomide (TMZ) is the standard chemotherapeutic agent for glioblastoma due to its ability to cross the blood-brain barrier. However, glioblastomas exhibit resistance to TMZ, decreasing its efficiency [163]. Cx43 activity has been identified as a key contributor to this resistance and is associated with poor prognosis [103], 164]. Preclinical studies have explored strategies to overcome Cx43-mediated resistance. In a rat glioblastoma model, an antibody targeting the second extracellular loop of Cx43 prevented the development of TMZ resistance [104]. *In vitro* glioblastoma cell lines treated with the Cx43 αCT1 peptide, which prevents Cx43 activation by inhibiting phosphorylation at the Ser368 site on the Cx43 cytoplasmic tail, exhibited reduced PI3K/AKT survival signaling and increased apoptosis. However, due to the short half-life of αCT1, co-treatment with the selective PI3K inhibitor TGX-221 (targeting the p110-β subunit that interacts with Cx43) was proposed. This combination therapy successfully reversed TMZ resistance in glioblastoma cell lines and in xenografted mice [103]. Since Cx43 activity also enhances glioblastoma invasiveness (178), therapeutic approaches using antibodies or peptides to specifically target Cx43 represent promising avenues to improve treatment efficacy.

## Summary and conclusions

Connexin dysregulation contributes to the pathophysiology of CNS disorders and has therefore emerged as a potential therapeutic target. However, the translational development of connexin-based therapies for neurodegenerative and neuroinflammatory diseases remains at an early stage. Most available evidence is currently derived from mechanistic and preclinical studies, with relatively few examples extending to clinical evaluation. Among the most studied approaches are connexin-targeting peptides and hemichannel inhibitors designed to limit the release of pro-inflammatory and neurotoxic factors. Compounds such as TAT-Gap19 and INI-0602 are of interest because they selectively inhibit pathological hemichannel activity while aiming to preserve gap junctional communication; however, clinical evidence remains limited and indirect. In addition, Modafinil, which has been reported to enhance astroglial gap junction communication, has shown modest benefit in PD studies, although the contribution of connexin modulation to these effects remains incompletely defined. Additional strategies, such as Cx43^+^ exosome-based therapies, are also under preclinical investigation, but substantial work is required to determine their specificity, safety, and delivery potential before clinical translation can be considered.
